# The tpe - teresa project: Enhancing long-term management in obesity and binge eating disorder

**DOI:** 10.1192/j.eurpsy.2021.961

**Published:** 2021-08-13

**Authors:** E. Prosperi, G. Guidi, M.F. Calabria, L. Gnessi, L. Iocchi

**Affiliations:** 1 Siet, Italian Society Therapeutic Education, Roma, Italy; 2 Sapienza University, Rome, Italy, Dipartimento di Medicina Sperimentale, Roma, Italy; 3 Sapienza Università Di Roma, Italy, Dipartimento di Ingegneria Informatica Automatica e Gestionale, Roma, Italy

**Keywords:** therapeutic education, obesity, binge eating disorder, assistive technology

## Abstract

**Introduction:**

Therapeutic Education (TE) is a powerful tool in the multidisciplinary intervention to improve lifestyle and acquire management skills for chronic diseases, including obesity, a clinical condition whose cure is highly threatened by low long-term adherence to therapeutic recommendations. The urgent need to promote persistent lifestyle change and concordance to treatment in PwO is globally recognized. TE programs offer a vast number of long-term management skills, but it yet deals with a consistent drop-out rate, and we believe that Assistive Technologies (AT) can become a powerful tool to boost independence and improve participation.

**Objectives:**

The goal of our study was to devise and validate an innovative multidisciplinary approach to obesity and binge eating disorder, based on the sinergy between the medical-psychological field and assistive techonolgy.

**Methods:**

We developed “TERESA” (Therapeutic Educational Robot Enhancing Social interActions) (fig. 1), a social humanoid robot, and implemented it to collaborate in a TE programs in order to enhance social interactions, improve knowledge acquisition and adherence to treatment. The specific TE intervention, called Education towards Choice and Awareness, was based on 3rd generation cognitive-behavioral approaches and consisted in eight informative and experimental meetings.

**Figure:**
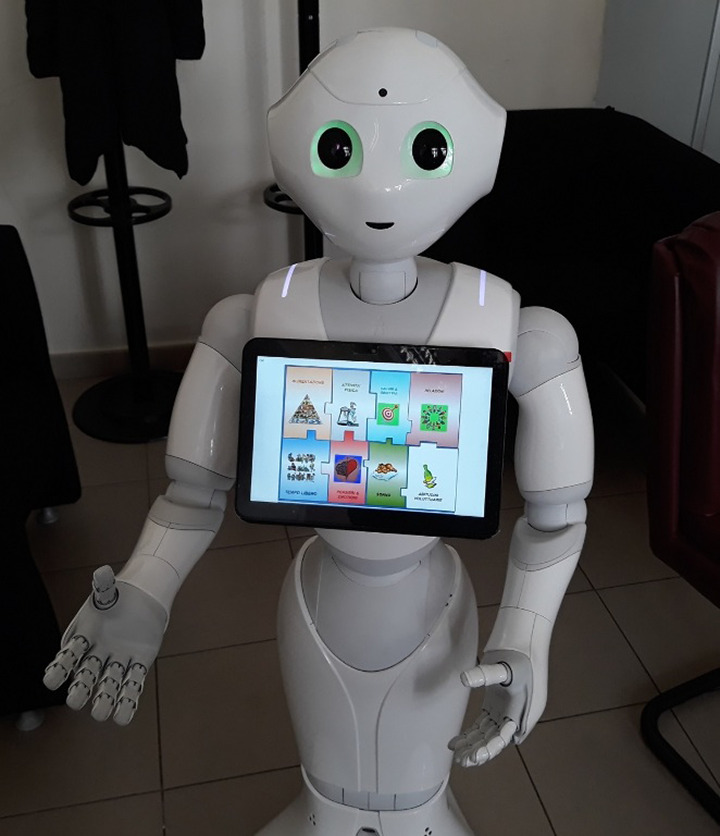

**Results:**

Taking part in the TE-TERESA integrated protocol determined and improvement in psychopathological domains (anxiety, negative mood, quality of life) and a stronger concordance to the therapeutic protocol.

**Conclusions:**

Our research paves the way for the clinical use of Assistive technology (AT), highly promoted by the WHO to help people with numerous disabling clinical conditions improve their quality of life and acquire self-management skills.

